# Event and Comment

**Published:** 1913-05-15

**Authors:** 


### DENTAL REGISTER.

**Vol. LXVII. May 15, 1913. No. 5**

### EVENT AND COMMENT.

PROFESSIONAL VERSUS COMMERCIAL IDEALS. We are reprinting in this issue an address delivered by the eloquent Canadian orator, Dr. A. W. Thornton, at a banquet in Pittsburgh, which strikes a timely blow at the prevalent effort and tendency of some dental writers to blind our younger men to the nobler ideals of a professional calling by holding out the fascinating and alluring possibilities of material aggrandizement. The address is not only timely but its tone is tremendously wholesome, because it calls attention to the true ideals which ought to prevail in every professional calling, where not only the good health but the moral and material welfare of our patients is such a strong and significant matter of consideration. Dr. Thornton makes clear the point that a professional service deserves a proper recompense when practicable, but that the fee to be earned is in no sense to be the controlling or even influential factor as to the manner or quality of the service to be rendered. The propaganda for better knowledge of the actual cost of professional services is important from an educational standpoint and may be of great assistance in the equitable adjustment of fees, but its importance is in no sense comparable to the cultivation of skill and to the acquirement of knowledge which will increase one's ability to render a more adequate and satisfactory service. Read Thornton's address and carefully consider the thought he brings out and see if it does not throw a brighter light on the nobility of our calling.

DENTAL LEGISLATION. We seem to be having a sort of epidemic of new legislation to safeguard the interests of the people in the matter of dental health, at least a half dozen states have enacted new laws or amended former laws during the past winter. The item which seems to be securing the most attention is better education of the dentist. The favorite plan seems to be to require better preliminary education for admission to the schools and a more completely administered curriculum for the colleges and more effective and stringent tests of the college product by the State Board of Examiners. There are still a few states in which any candidate, whether a graduate or not, will be examined and registered if found efficient, but the large majority of the states decline to even examine an undergraduate. Just what is the cause of this movement we are not prepared to discuss, but it is not unlikely that it results from the work of the National Board of Examiners rather than from any professional agitation. This ought to have a wholesome and beneficial effect, because the Examiners are in a position to determine with a fair degree of accuracy the shortcomings of our educational system. Because of their knowledge of the subject and their moral and legal responsibilities to the profession and the public, their duty is made plain and imperative. We notice in all these laws a tendency to put under legal surveillance the practicing dentist, who heretofore when once registered, was at liberty to choose his own methods or ideas of practice, whether these ideas were calculated to conserve the public health best or not. In some of the new laws permanent registration is supplanted by annual registration, which gives the State Boards a chance to decline a reissue of a new license to a practitioner who is grossly immoral or incompetent. We have no doubt such a provision faithfully carried out will do much to restrain and correct a growing tendency to selfish and unfair professional services.

In the State of Massachusetts a strong effort is being made to get an official recognition for an office assistant under the name of a trained nurse. The propriety of this movement is seriously questioned, and it is doubtful if this measure will be made a law in any state in the near future.

In several states a distinct advance has been secured in the matter of providing, through nominations, by the State Dental Society of twenty or thirty capable men from whom the Governor shall select his appointees to the office of Examiner. This ought to eliminate the political scramble for office and should secure better men for our State Boards in the near future.

The new Michigan Law is a very good illustration of this modern tendency, and it is being copied by other states. A new law for Arizona makes a closer alliance with the state officials, especially in regard to the financial administration and prosecution of unlicensed dentists and the revocation of licenses. We believe this a proper step and other departments of the work might be profitably given a closer affiliation with the state executive officials than obtains at present. We have hoped for many years that the various state laws might become more uniform in requirement, in order that reciprocal relations could be more equitably established.

